# MINDS: A Translator to Embed Mathematical Expressions Inside SPARQL Queries

**DOI:** 10.1007/978-3-030-59833-4_7

**Published:** 2020-10-27

**Authors:** Damien Graux, Gezim Sejdiu, Claus Stadler, Giulio Napolitano, Jens Lehmann

**Affiliations:** 8 Linköping University, Linköping, Sweden; 9 University of Amsterdam, Amsterdam, Noord-Holland The Netherlands; 10 Department of Computer Science, Vrije Universiteit Amsterdam, Amsterdam, Noord-Holland The Netherlands; 11 St. Pölten University of Applied Sciences, St. Pölten, Austria; 12 FIZ Karlsruhe – Leibniz Institute for, Karlsruhe, Germany; 13 Karlsruhe Institute of Technology, Karlsruhe, Germany; 14 UAS St. Pölten, St. Pölten, Niederösterreich Austria; 15 Vienna University of Economics and Business, Vienna, Wien Austria; 16 VU Amsterdam, Amsterdam, The Netherlands; 17 ADAPT Centre, Trinity College Dublin, Dublin, Ireland; 18 ADAPT SFI Centre, Trinity College Dublin, Dublin, Ireland; 19 Deutsche Post DHL Group, Bonn, Germany; 20 Leipzig University, Leipzig, Germany; 21 Fraunhofer IAIS, Sankt Augustin, Germany; 22 Smart Data Analytics, Bonn University, Bonn, Germany

## Abstract

The recent deployments of semantic web tools and the expansion of available linked datasets have given users the opportunity of building increasingly complex applications. These emerging use cases often require queries containing mathematical formulas such as euclidean distances or unit conversions. Currently, the latest SPARQL standard (version 1.1) only embeds basic math operators. Thus, to address this shortcoming, some popular SPARQL evaluators provide built-in tools to cover specific needs; however, such tools are not standard yet. To offer users a more generic solution, we propose and share MINDS, a translator of mathematical expressions into SPARQL-compliant bindings which can be understood by any evaluator. MINDS thereby facilitates the query design whenever mathematical computations are needed in a SPARQL query.

## Introduction

During the past two decades, semantic web technologies for the web have been developed and it is now possible to produce, share, analyze and interlink large knowledge graphs (sometimes containing billions of facts) structured using the RDF w3c standard [12]. Additionally, the W3C has standardized SPARQL [14], the *de facto* query language dedicated to RDF which has been more recently improved to add new features, see *e.g.* [19] for its current version. Furthermore, several projects have been created where SPARQL public endpoints are openly available to access data such as DBpedia [9] or YAGO [16]. As a consequence, to leverage these resources the Semantic Web community has been developing more and more complex use cases involving several endpoints which are then queried together using federated SPARQL queries to build or extract knowledge from combinations of multiple endpoints. In addition, these use cases sometimes require the computation of mathematical formulas which combine values according to specific patterns, to either filter or return the results. However, in the current version of the standard^1^, only the four basic mathematical operators are available ($+,~-,~*,~/$) and some basic predefined functions, such as CEIL or FLOOR. To address this lack in the standard, some popular evaluators allow extensions to the SPARQL language to cover popular mathematical functions (*e.g.* trigonometric operations). Nonetheless, this results in queries especially built to be executed in a specific system and which therefore cannot be shared among users.

To gain in interoperability, we propose and share MINDS: a translator to embed Mathematical expressions INsiDe S parql queries. Our implementation is openly available under the terms of the *Apache License version 2.0* from:

https://github.com/SmartDataAnalytics/minds

MINDS translates the given mathematical expressions into a list of SPARQL-compliant bindings *i.e.* BIND((...)AS ?var). This approach allows thereby the obtained SPARQL queries to be executed by any kind of evaluator while facilitating the task of query design.

The rest of this article is structured as follows. First, we review the related work in Sect. 2 and next, we motivate our approach with an example requiring mathematical formulas in Sect. 3. Then, we describe MINDS in Sect. 4, before presenting in Sect. 5 some accuracy results about our methods and some comparisons against existing SPARQL evaluators. In Sect. 6, we present various use cases implying the use of MINDS. Finally we conclude in Sect. 7.

## Related Work

In this section, we provide an overview of the related work regarding mathematical formulas inside SPARQL queries. Due to the SPARQL standard lacking the specification of something essential as basic math functions^2^, different approaches have emerged to serve this need.

In fact, some SPARQL evaluators do not give the possibility of computing mathematical functions inside queries at all. This is for instance the case with 4store [7], RDF3X [13] or SPARQLGX [5] which are nonetheless popular evaluators from the literature renown for their performance. However, arguably, the research focus of these systems was on optimization of joins and indexes and less on feature completeness.

Currently, all practical relevant SPARQL evaluators offer the opportunity of computing mathematical functions inside the BIND elements and projections. While the SPARQL standard defines the built-in functions as part of the syntax^3^, the widely adopted approach by evaluator developers is to take advantage of the Function Call rule, which allows arbitrary IRIs to be used as function names. Hence, function extensions typically require no changes to the SPARQL syntax. However, the lack of standardization implies two drawbacks:

- Firstly, the namespaces, local names and signatures of functions may vary between SPARQL engines, which makes it tedious –if not prohibitive– to exchange backends.
- Secondly, the means of computation of a function and therefore the results may differ between evaluators.

All popular SPARQL evaluators –often used to serve public endpoints– such as Virtuoso [4], Jena-Fuseki [8], GraphDB^4^ and Stardog^5^ feature mathematical functions, yet, using different IRIs. For instance, Virtuoso uses the bif: namespace, whereas Stardog reuses the XPath function namespace^6^. Using such an approach of naming differently similar function/operator^7^ implies a loss of interoperability, especially, it make the design of federated SPARQL queries far more complex. Finally, some evaluators implement GeoSPARQL [2] giving then access to spatial functions for use in SPARQL queries such as finding a distance or computing a convex hull.

Compared with existing evaluators which provide sometimes built-in mathematical functions, MINDS chooses to use approximations when necessary in order to remain fully compliant with the SPARQL language.

## Motivating Example

To have a better understanding of when mathematics may be needed in SPARQL queries, we consider a use case based on the geographical position of fossils found. Having a dataset recording the found fossils, we want to list the fossils:

- found in the last ten years;
- located 100 km around a specific position;
- older than 1 000 years.

For clarity reasons, we will consider a simplified dataset recording Cartesian positions, a ${}^{14}$C-ratio and the discovery year. Each fossil is then represented by an identifier using the following structure: 
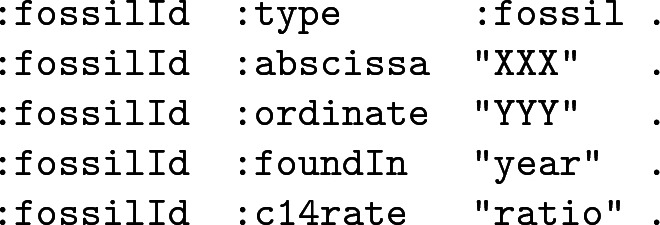
 As a consequence, to list all the fossils, one might run this SPARQL query: SELECT ?f WHERE { ?f :type :fossil}. In the rest of this Section, we will refine step by step this query to add the restrictions specified above, emulating the process of a query designer.

**a – Found in the last ten years.** This constraint implies the filtering of the records according to the year of their discovery. Considering that the current year is 2020, we will keep only fossils found after 2010 and we can ask: 
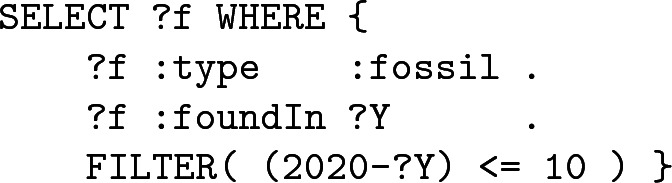
 In this particular case, only a simple FILTER (involving a simple operation) is required to refine the join.

**b – 100 km around a position.** Then, we want to return fossils found around a specific position whose Cartesian coordinates are (Px,Py). To do so, we have to compute Euclidean distances between this position and the fossils using the classic formula: $d=\sqrt{\varDelta {}x^2 + \varDelta {}y^2}$. However, according to the standard, there is no square operator and no square-root. Obviously, we can escape from these issues easily by comparing $d^2$ instead of *d*. Our SPARQL query thus becomes: 
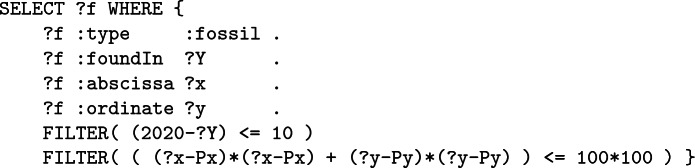
 As one can see, the FILTER condition is getting longer –increasing the probability of errors and typos for example– and in this example we only deal with simplified data (for instance no unit conversions are needed).

**c – Older than 1 000 years.** The last condition only retains fossils which are older than 1 000 years. However, the considered dataset does not share ages but instead ${}^{14}$C-ratios *r* of fossils which can be used using radiocarbon dating –considering the ${}^{14}$C half-life $t_{1/2}$ *i.e* 5 700 years– to find the age *t*(*r*) according to the following formula:

$$\begin{aligned} t(r) = \left( \frac{\ln (r)}{-0.693} \right) ~.~t_{1/2} \end{aligned}$$

This expression involves the natural logarithm which is, however, not part of the standard. Therefore, to compute this expression, the query designer has to approximate the logarithm, using for example a decomposition in series:

$$\begin{aligned} \forall y \in ]0,+\infty [, ~ \ln (y)=2\sum _{k=0}^{+\infty }\frac{1}{2k+1}\left( \frac{y-1}{y+1}\right) ^{2k+1} \end{aligned}$$

The FILTER can now by written:  where ?LOG is a variable embedding the logarithm approximation whose result quality depends on the number of terms used in the decomposition. Considering only the first three terms ($k\in [0,2]$) and the ${}^{14}$C-ratio ?rate of fossils, we have: 
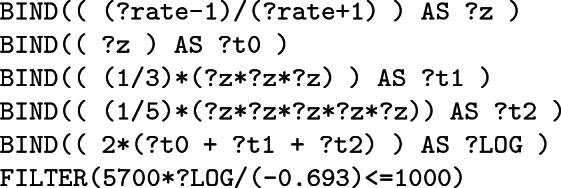


As a consequence, it appears that building this simple approximation for its first three terms already leads to a rather complicated query.

**Furthermore.** As stated previously, the example has been simplified for the sake of clarity. Firstly, the series approximation should indeed involve more terms *i.e.* at least 5 (see Sect. 5 for more details about the approximation preciseness). Secondly, when dealing with geographical data on Earth, latitude and longitude coordinates are actually preferred to Cartesian ones. Thus, considering two points $P_1(lat_1,lon_1)$ and $P_2(lat_2,lon_2)$, the distance *d* should be calculated using the Haversine formula to calculate the great-circle distance:Thereby, to compute *d* with this formula, several non-standard functions are required: **7** trigonometric ones and **2** square-roots. If this very query were to be evaluated, the designer would have to write herself the multiple decompositions in series which would be tedious and a possible source of errors. In the next Section, we introduce MINDS: our solution to help query designers when dealing with mathematical expressions.

**Fig. 1.**
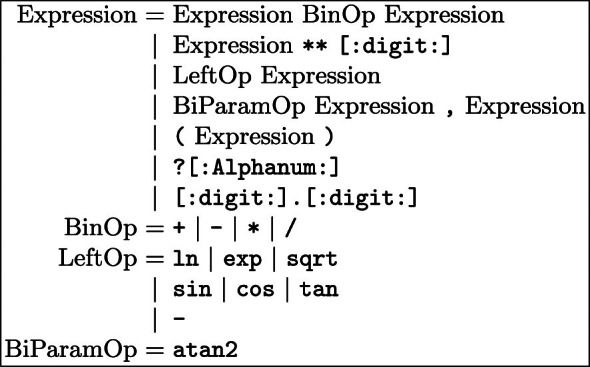
Grammar of the expressions understood by MINDS.

## MINDS: From a Math Formula to SPARQL Bindings

To tackle this gap in the SPARQL standard, and to help query designers in their tasks, we developed a software called **MINDS**. In a nutshell, it allows users to input a mathematical expression and obtain –only using standard operators and keywords– the exact corresponding translation, or an approximation if a decomposition in series has to be involved.

Practically, we developed MINDS as an external software which can be run when designing queries. It is written in Python [18] and its core currently represents about 500 lines of code. Technically, the given formula is parsed using a dedicated implementation of the popular Lex and Yacc tools [11] for Python named PLY^8^. Then, once the formula is split into tokens, the translating rules are applied recursively to generate the final result. For instance, considering again the example of Sect. 3, the “2020-?Y” expression will be translated into:

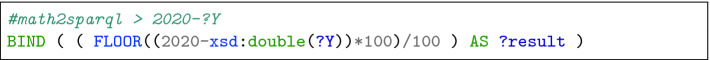



Compared to the solution presented in Sect. 3, the actual binding is already more complicated: first, since it specifies that ?Y should be considered as a double; and second, since it truncates the result to keep only two digits of precision with the FLOOR keyword of the standard. Actually, this precision parameter can be set by the user in MINDS, for instance to 5:

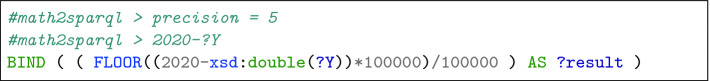



Therefore, MINDS is still relevant to handle even simple expressions that are cumbersome to express in SPARQL such as the $d^2$ (*i.e.* a squared Euclidean distance) of the previous Section:

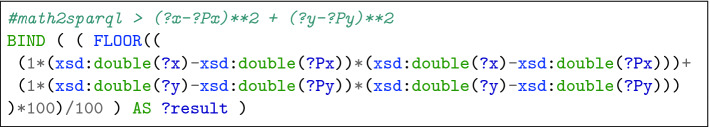



**Fig. 2.**
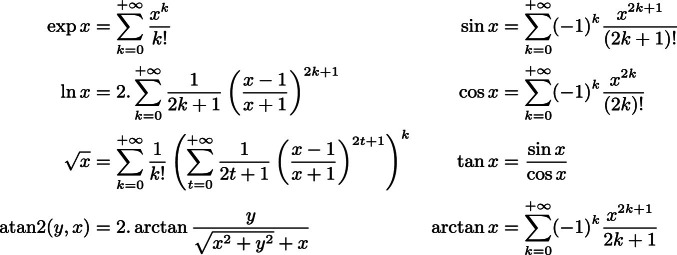
Series currently used by MINDS.

We furthermore describe in Fig. 1 the grammar which is understood by MINDS. In particular, our translator is able to deal with the four basic operators of SPARQL (*i.e.* + - * /) extended with the power operator (** in MINDS) while respecting conventional priorities. Moreover, our solution also provides several translation rules to deal with mathematical functions *e.g.* trigonometric functions and even with functions of multiple variables *e.g.* . Nonetheless, these additional functions are not part of the standard and have to be expressed only using allowed SPARQL operators: MINDS is then able to compute approximations to translate into bindings these functions. Indeed, it uses when necessary a series decomposition such as the ones listed in Fig. 2 and technically a new binding is generated for each series so that the query evaluator might be able to store the sub-result. For instance, considering $x^2 + \exp (y+3z)$ which involved the computation of the exponential of a linear expression, MINDS returns:

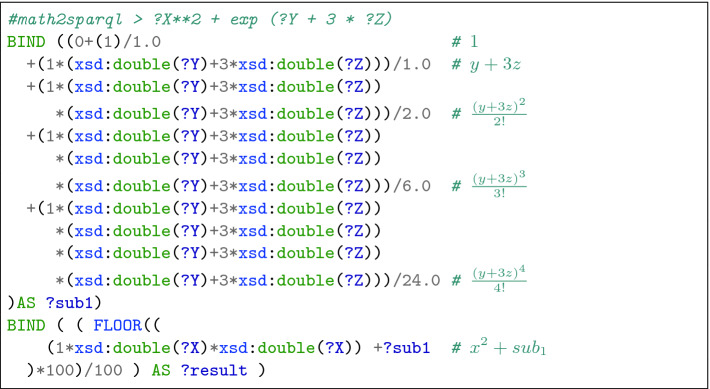



As expected, MINDS automatically converts the exponential part into an approximation using the classic series of the exponential (see Fig. 2 for more details); in this case only the first five terms of the series where considered. As it will be described in Sect. 5, the more terms are involved the more precise the results will be; nonetheless, it is also important to mention that MINDS allows query designers to choose as a parameter this number of terms. Moreover, it is able to understand any kind of combination using its recognized keywords and it generates recursively the sub-bindings when necessary.

**Fig. 3.**
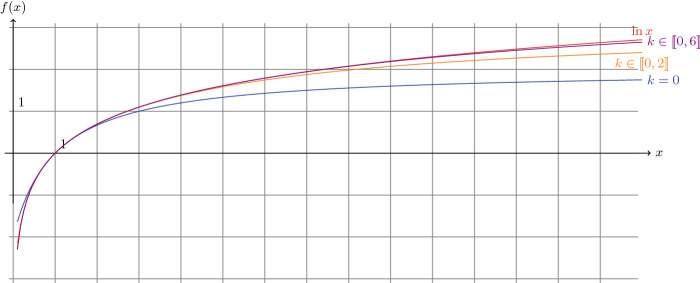
Natural logarithm and its approximations.

**Fig. 4.**
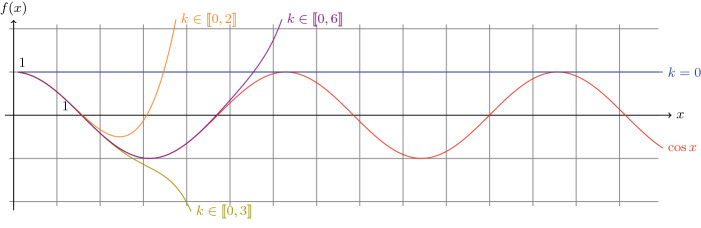
Cosine and its approximations.

## Precision Results

First of all, we want to pinpoint that the bindings generated by MINDS offer the same orders of magnitude as the tested built-in functions in terms of execution times. Indeed, the evaluation of a BIND expression or the call to an internal method are both executed in sub-second times; for more details, we refer the reader to the end of this Section, where external links of running queries on various SPARQL endpoints are available.

**Fig. 5.**
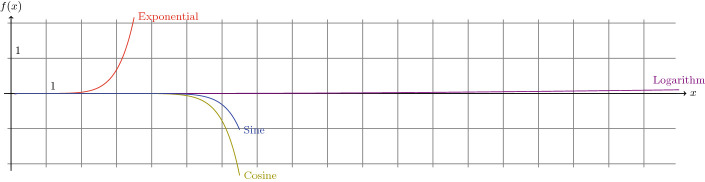
Approximation drifts (first seven terms) from the theoretical functions.

Moreover, since MINDS uses approximations based on series for some mathematical functions (see Fig. 2 for details), we further describe in this Section the accuracy of such a method before comparing MINDS with built-in functions of popular SPARQL endpoints.

**Accuracy.** First, we should remind that the number of terms used in the series has an impact on the quality of the approximation. Here, we review the approximation of the natural logarithm $\ln$ in Fig. 3, and of the cosine $\cos$ in Fig. 4. In both cases, we draw the exact function as a reference in red, together with several approximations: in blue only the first term of the series, in orange the first three ones and in purple the first seven ones. Thereby, it is evident that by considering **only** the first seven terms already provides more than 95% of accuracy for the logarithm in the interval [1,20] and the approximation for $\ln (100)$ is still 80% accurate. However, with trigonometric functions (see *e.g.* the cosine in Fig. 4), more terms are required. Nonetheless, to tackle this problem, MINDS takes advantage of the periodicity of these functions and actually:

1. adds an additional binding to represent an approximated value of $2\pi$ *i.e.* BIND((6.28318530718) AS ?2P);
2. replaces the expression ?f inside the $\sin$ or the $\cos$ function with the remainder of the division of ?f by $2\pi$ *i.e.* (?f - ?2P * FLOOR(?f/?2P)).

This method allows MINDS to stay within an interval in which the accuracy remains above 80% with the first seven terms. More generally, in Fig. 5, we present the drifts between mathematical functions and their respective approximations using the first seven terms of their series. This representation allows the query designers to determine the intervals where the proposed approximations of MINDS still have an accuracy above a chosen threshold, letting them decide the appropriate number of terms in the series to be generated.

**Fig. 6.**
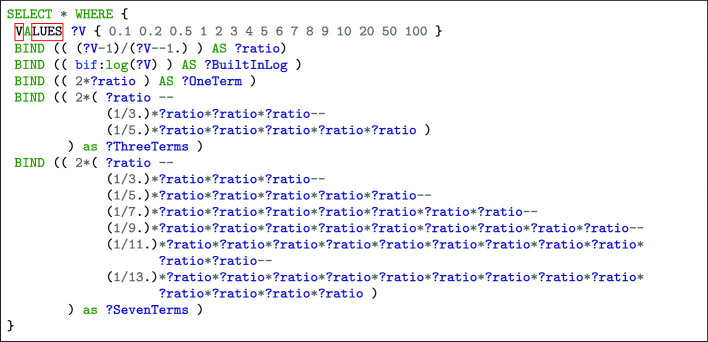
Query involving Virtuoso’s built-in *ln* and approximations for different values.

**Comparison with Built-in Functions.** Since mathematical functions are not part of the SPARQL standard [19], most of the popular systems providing endpoints have implemented their own versions of some functions (see Sect. 2 for more details about these systems). In this study, we also present comparisons between MINDS approximations and the built-in functions from some of these systems, namely: Virtuoso^9^ [4], GraphDB^10^ and JenaFuseki^11^ [8].

In Table 1, we present the raw results of a SPARQL query which computes on Virtuoso for several values (?V): the built-in natural logarithm (?BuilInLog) using the bif: prefix and three bindings generated by MINDS varying the number of terms involved *i.e.* one, three and seven (see Fig. 6). We observe that the accuracy measured corresponds to the one expected theoretically (as drawn *e.g.* in Fig. 3 and 4). This observation implies that the Virtuoso engine executes exactly the operations listed in the bindings (without rounding nor truncating).

More generally, since the built-in functions are specific addons provided by the systems, the set of available mathematical functions may vary across them: for instance, GraphDB provides very specific functions such as “*hypot*(*x*, *y*)” which returns $\sqrt{x^2+y^2}$ or “*IEEEremainder*(*x*, *y*)” which is the remainder operation on two arguments as prescribed by the IEEE 754 standard. Furthermore, currently (without MINDS), the query designers have to tune their SPARQL queries for each evaluation engine. For example, we list here various syntaxes to evaluate a logarithm:

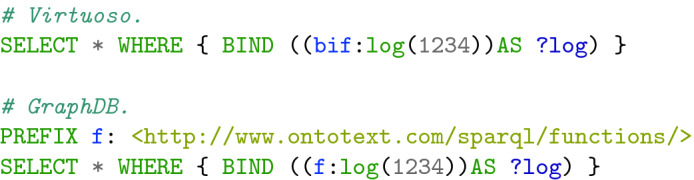

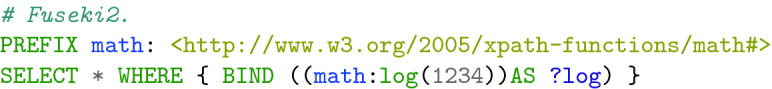





Notice that it is possible to directly run these examples –based on the natural logarithm^12^– on several systems, considering that these systems are used to provide public SPARQL endpoints by a number of popular services, some of which available at the following links:

- **Virtuoso** on the DBpedia endpoint  ;
- **GraphDB** on the FactForge endpoint  ;
- **Fuseki2** on the ZBW Labs endpoint  .

The three above hypertext links provides visualizations of the SPARQL queries and automatically compute and display the results. They provide similar results as the ones already presented in Table 1.

**Table 1.** Virtuoso’s built-in natural logarithm vs some MINDS bindings.

?V	?BuiltInLog	?OneTerm	?ThreeTerms	?SevenTerms
0.1	$-2.30259$	$-1.636363636363636$	$-2.148161762423083$	$-2.28612550677627$
0.2	$-1.60944$	$-1.333333333333333$	$-1.583539094650206$	$-1.608934294900188$
0.5	$-0.693147$	$-0.666666666666667$	$-0.693004115226337$	$-0.693147170256012$
1	0.0	0	0	0
2	0.693147	0.666666666666667	0.693004115226337	0.693147170256012
3	1.09861	1	1.095833333333333	1.098607062425422
4	1.38629	1.2	1.375104	1.386202224193573
5	1.60944	1.333333333333333	1.583539094650206	1.608934294900188
6	1.79176	1.428571428571429	1.745899525991154	1.790187408711124
7	1.94591	1.5	1.876171875	1.942329693525345
8	2.07944	1.555555555555556	1.983078460261816	2.072740626022152
9	2.19722	1.6	2.072405333333333	2.186225968329208
10	2.30259	1.636363636363636	2.148161762423083	2.28612550677627
20	2.99573	1.80952380952381	2.545790028209391	2.880218635963087
50	3.91202	1.92156862745098	2.840323022038755	3.419833927257202
100	4.60517	1.96039603960396	2.950171566927436	3.64870669515376

## Use Cases

MINDSaims to be a generic tool which can be integrated into existing system for SPARQL parsing or mapping to different transformations. To this aim we are developing a number of use case implementations on different tools and systems. We group such use cases into two different categories:

***Integration***

*SPARQL–to–SQL rewriter – Sparqlify.* Sparqlify^13^ is a SPARQL-SQL rewriter that enables the definition of RDF views on relational databases and their querying using SPARQL [15]. MINDSis being used for mathematical transformations into SPARQL bindings embedded into Sparqlify. Users will write SPARQL queries following the instructions represented by MINDSand then Sparqlify will take over the query rewriter into SQL syntax.

*Semantic Analytics Stack – SANSA.* SANSA [10] is an open source^14^ *data flow processing engine* for performing distributed computation over large-scale RDF datasets. It provides data distribution, communication, and fault tolerance for manipulating massive RDF graphs and applying machine learning algorithms on the data at scale. SANSA uses Sparqlify as an underlying infrastructure for the integration of existing SPARQL-to-SQL rewriting tools. By doing so, it enables mathematical transformations as well via MINDSas a support add-on.

***Usability***

*Blockchain – Alethio Use Case.* Alethio^15^ is modeling an Ethereum analytics platform that endeavors to provide transparency over the transaction pool of the Ethereum network. Their 5 billion triple dataset contains large scale blockchain transaction data modelled as RDF according to the structure of the Ethereum ontology^16^. Alethio has been using SANSA as a scalable processing engine for their large-scale data processing tasks, such as querying the data in real time via SPARQL and performing related analytics [6, 17]. MINDS was used through SANSA integration and served as an easy-to-use mathematical function evaluator, such as time-series of the latest exchange values, average transaction size or even filtering some chains considering geometrical-mean of some included parameters.

*Geospatial Data – SLIPO.* SLIPO^17^ was an EU Horizon2020 project which aimed at developing linked data technologies for the scalable and quality-assured integration of Big Point of Interest (POI) datasets [1]. SLIPO used SANSA as a scalable querying engine to deal with their large-scale POIs data [3]. In particular, SLIPO aimed at discovering areas of interests using POI datasets which implies, for instance, searching road segments where amenities with some common parameters are located. To do so, MINDS is being used there to filter POIs which are inside a convex hull.

## Conclusion

In this article we introduced MINDS^18^, a translator of mathematical expressions into SPARQL bindings. MINDS is also open source and shared on the Github platform which, in addition, provides us with the needed tools to manage an open-source software *i.e.* a bug tracker, a way to integrate external contributions or also a release generator. We do hope this tool will help query designers in their tasks by providing *in an instant* the SPARQL compliant translation of complicated mathematical expressions, while giving them the ability of adjusting parameters in approximations.

## Footnotes

1 https://www.w3.org/TR/2013/REC-sparql11-query-20130321/#expressions.

2 Currently, the *SPARQL 1.2 Community Group* which aims to advance SPARQL functionalities, is describing several mathematical operators that could be added in the next iteration of the standard. https://github.com/w3c/sparql-12/.

3 https://www.w3.org/TR/sparql11-query/#grammar.

4 https://ontotext.com/products/graphdb/.

5 https://www.stardog.com/.

6 https://www.w3.org/2005/xpath-functions/math#.

7 Implementations for built-in STDEV in Virtuoso, Fuseki, Stardog, Sesame: https://gist.github.com/albertmeronyo/c6ab285d0b73b05392e2f9b8a5bbea82.

8 Python Lex-Yacc repository: https://github.com/dabeaz/ply.

9 https://virtuoso.openlinksw.com/.

10 https://ontotext.com/products/graphdb/.

11 https://jena.apache.org/documentation/fuseki2/.

12 More examples online from https://smartdataanalytics.github.io/minds/ where some other built-in functions are reviewed with other sets of values.

13 https://github.com/SmartDataAnalytics/Sparqlify.

14 https://github.com/SANSA-Stack.

15 https://aleth.io/.

16 https://github.com/ConsenSys/EthOn.

17 http://www.slipo.eu/.

18 MINDS web-page: https://smartdataanalytics.github.io/minds/ which offers more information and details about the software such as for instance tutorials, running examples inside other SPARQL evaluators, accuracy charts.
